# Accelerated Epigenetic Aging in Children and Adults With a Fontan Circulation

**DOI:** 10.1016/j.jacadv.2024.100865

**Published:** 2024-02-17

**Authors:** Nigel E. Drury, John Stickley, Rami Dhillon, Thomas P. Gaffey, Wei Guo, Xiaojing Yang, Yap C. Chew, Paul F. Clift

**Affiliations:** a Institute of Cardiovascular Sciences, University of Birmingham, Birmingham, United Kingdom; b Birmingham Children’s Hospital, Birmingham, United Kingdom; c Queen Elizabeth Hospital Birmingham, Birmingham, United Kingdom; d Zymo Research Corporation, Irvine, California, USA; e Epimorphy LLC, Tustin, California, USA

The Fontan circulation represents a final common surgical palliation for those born with a single ventricle heart condition, whereby the functioning ventricle is used to support the systemic circulation with passive pulmonary blood flow in series. However, with elevated central venous pressure and reduced cardiac output, this inherently inefficient circulation predisposes to multiple morbidities, including progressive functional decline, neurocognitive impairment, sarcopenia, osteoporosis, renal dysfunction, and liver fibrosis.^1^^,^^2^ These clinical manifestations, typically associated with frailty and older age, are consistent with premature aging occurring in their third and fourth decades, leading to premature death.^2^ It therefore remains a life-limiting condition with few late treatment options in a rapidly growing adult population.

Epigenetic clocks, based on the extent of DNA methylation at multiple 5’-cytosine-phosphate-guanine-3’ (CpG) sites, have been used to estimate epigenetic age, which is tightly correlated with chronological age in human controls.^3^ In this proof-of-concept study, we evaluated whether the Fontan circulation is associated with accelerated epigenetic aging.

Ethical approval was obtained from the North-West-Haydock NHS Research Ethics Committee (20/NW/0001, February 20, 2020). Patients on a single ventricle pathway under follow-up in Birmingham, UK were recruited, excluding any with known chromosomal or genetic disorders. With written informed consent, clinical data and blood were obtained at routine clinic visits.

The reference cohort was comprised of 421 healthy volunteers (n = 228, 54.2% male), without known age-related disease, with a mean chronological age of 35 years (IQR: 28-49 years).

Whole blood samples were collected, immediately preserved in EDTA or DNA/RNA Shield, and stored at −80 °C. Sample DNA was extracted, purified using *Quick*-DNA Miniprep Plus Kit (ZymoResearch), and quantity/quality control checks performed. Bisulfite conversion was performed using EZ DNA Methylation-Lightning kit (ZymoResearch) according to protocol. Bisulfite-converted DNA libraries containing >2,000 age-associated CpG loci were prepared for Simplified Whole-panel Amplification Reaction Method (SWARM) and sequenced using Illumina NovaSeq technology (Illumina) for >1,000 × coverage.

Sequence reads were identified using Illumina base-calling software and aligned to the reference genome using Bismark, an aligner optimized for bisulfite sequencing and methylation calling. The methylation level of each sampled cytosine was estimated as the number of reads reporting C, divided by the total number of reads reporting C or T. Calculated DNA methylation values were used to assess DNA methylation age (DNAge) according to Zymo’s proprietary algorithm, blinded to clinical data including chronological age.

Analysis was performed using R v3.6. ΔAge was calculated as the difference between chronological age (date of sample − date of birth) and estimated DNAge. An unpaired *t*-test was used to compare groups, with statistical significance at *P* < 0.05.

Blood was obtained from 40 patients (n = 25, 63% male): 10 children (median 10.3 years, IQR: 9.4-13.4 years) and 30 adults (median 29.8 years, IQR: 24.0-35.3 years) with Fontan circulation. Twenty-six (65%) had a systemic left ventricle, 21 (53%) had an extra-cardiac conduit, and median systemic oxygen saturations were 94% (IQR: 90%-95%); of the adults, 27 (87%) were NYHA functional class I-II.

**Figure 1:**
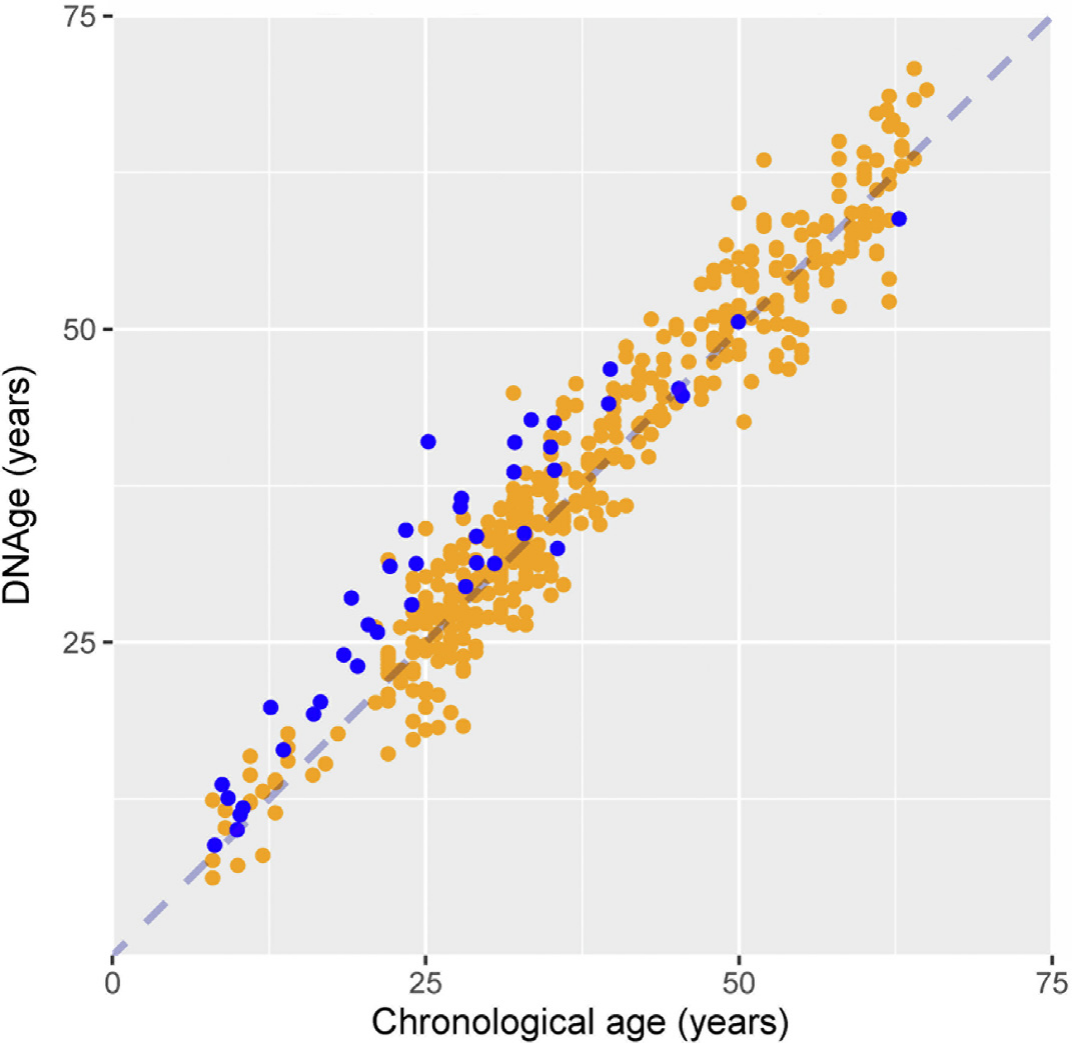
**Relationship Between Chronological Age and Estimated DNAge in Fontan Patients and Reference Cohort** Relationship between chronological age and estimated DNAage in Fontan patients (n = 40) (blue circles) and reference count (n = 421) (orange circles).

Mean Δage post-Fontan was +4.4 ± 4.0 years overall, +2.8 years (SD 2.2 years in the children, and +4.9 ± 4.3 years in the adults, compared with +0.6 ± 3.6 years in the reference cohort (+0.6 SD, 2.5 in 21 children; +0.6 SD, 3.6 in 400 adults). Δage was significantly higher post-Fontan than in controls (*z* = 5.5, *P* < 0.0001), and in males (+5.6 ± 3.6 years) vs females (+2.4 ± 3.9 years) post-Fontan (95% CI: 0.8-5.7, *P* = 0.011). The relationship between DNAge and chronological age for patients and controls is shown in Figure 1. Only 3 patients had negative Δage; all were female with chronological age >35 years.

This study is the first to demonstrate that the Fontan circulation is associated with accelerated epigenetic aging, mirroring the premature aging clinical phenotype. Our findings suggest that this process occurs in childhood and continues during adult life, potentially due to the chronic systemic stress of Fontan physiology. The mean Δage of +4.9 years in our adult cohort is consistent with other multisystem conditions associated with premature aging, such as adults living with chronic human immunodeficiency virus infection (+5.2 years).^4^ ΔAge was higher in males than females, consistent with the finding that male sex is an independent predictor of late Fontan outcomes.^1^

As a pilot study, we had insufficient data to determine the relationship between chronological age and Δage; the equivalence seen in older patients may represent sampling bias or survivorship bias in those undergoing Fontan completion over 30 years ago, when late survival was rare. A larger clinical-epigenetic dataset could also assess the relative impact of morphological or clinical factors, such as systemic ventricle, Fontan type, atrial fenestration, and functional status, on accelerated epigenetic aging, while tissue-specific epigenetic profiling, eg, liver biopsies, may add value as a marker of organ injury and prognosis.^3^ Finally, we did not evaluate whether accelerated epigenetic aging is specific to Fontan physiology or present in other severe congenital heart defects.

There are currently limited options to improve Fontan durability during adulthood. While a causal role between DNA methylation and biological function remains controversial,^3^ targeting epigenetic aging may represent a novel treatment opportunity to delay phenotype progression. Recent studies suggest that partial epigenetic reprogramming using Yamanaka factors can modulate DNA methylation and potentially reverse the cellular effects of aging.^5^ This may have implications for patient management, including routine monitoring for early signs of aging-related morbidities.

**What is the clinical question being addressed?**

Is the Fontan circulation associated with accelerated epigenetic aging?

**What is the main finding?**

Patients with a Fontan circulation demonstrate accelerated epigenetic aging, which may provide a novel treatment opportunity to reduce the clinical effects of premature aging.
